# Simultaneous Measurement of Strain and Temperature Distributions Using Optical Fibers with Different GeO_2_ and B_2_O_3_ Doping

**DOI:** 10.3390/s23031156

**Published:** 2023-01-19

**Authors:** Shinsaku Hisada, Utanori Kodakamine, Daichi Wada, Hideaki Murayama, Hirotaka Igawa

**Affiliations:** 1 Japan Aerospace Exploration Agency, Tokyo 181-0015, Japan; 2 Graduate School of Frontier Sciences, The University of Tokyo, Chiba 277-8561, Japan

**Keywords:** optical fiber, fiber Bragg grating, distributed measurement, strain and temperature, error propagation

## Abstract

Compensating for the effects of temperature is a crucial issue in structural health monitoring when using optical fiber sensors. This study focused on the change in sensitivity due to differences in GeO${}_{2}$ and B${}_{2}$O${}_{3}$ doping and then verified the accuracy when measuring the strain and temperature distributions simultaneously. Four types of optical fiber sensors were utilized to measure the strain and temperature in four-point bending tests, and the best combination of the sensors resulted in strain and temperature errors of 28.4 μϵ and 1.52 °C, respectively. Based on the results obtained from the four-point bending tests, we discussed the error factors via an error propagation analysis. The results of the error propagation analysis agreed well with the experimental results, thus indicating the effectiveness of the analysis as a method for verifying accuracy and error factors.

## 1. Introduction

Optical fiber sensors are suitable for structural health monitoring (SHM) due to their advantages, such as their small diameter, light weight, and immunity to electromagnetic interference [1], and their durability is being studied for practical use [2]. In SHM with optical fibers, the strain of the structure is crucial. However, because optical fibers are sensitive to both strain and temperature, it is necessary to compensate for the effects of temperature variation due to the surrounding environment when obtaining the mechanical strain. Therefore, various methods of separating the effects of strain and temperature have been studied, such as the superimposed fiber Bragg grating (FBG) [3], hybrid FBG [4], superstructure FBG [5], FBG with different diameters [6], single-mode fiber-based up-taper–down-taper–up taper (UDU) structure [7], sidelobe power of the FBG [8], and tilted FBG [9,10]. In SHM, distributed measurements are particularly beneficial. Previous studies on the simultaneous measurement of the strain and temperature distribution included the combination of Brillouin optical time-domain analysis (BOTDA) and Rayleigh optical frequency-domain reflectometry (OFDR) [11]. With this method, an area of 92 m could be measured with a spatial resolution of 50 cm. Our group developed a method that used FBGs inscribed in a polarization-maintaining and absorption-reducing fiber (PANDA fiber) with OFDR [12,13].

Another important topic in the research on fiber-optic sensing systems is the correct and efficient evaluation of measurement errors. One error evaluation method involves developing mathematical models of the sensing system. Soto et al. evaluated the accuracy of a BOTDA sensing system by mathematically modeling a pump and probe light incident on an optical fiber and the measured Brillouin scattering spectrum and by considering a normal distribution of the signal noise [14]. Murayama et al. mathematically modeled an interference signal from an FBG in an OFDR–FBG system and compared the center wavelengths detected in simulations and experiments [15]. For sensing systems other than those containing optical fibers, previous studies adopted error propagation analysis to evaluate measurement errors. Mekid et al. calculated the measurement accuracy of a laser-doppler-based non-contact sensor by considering the error propagation from the error amount in each error factor, such as the wavelength of the laser and the beam-crossing angle [16]. Other examples included the calculation of the position measurement error in a 3D system [17] and the calculation of the error in snow accumulation estimation by using a ground-based laser scanner [18].

Here, we propose a method that involves the simultaneous measurement of the strain and temperature distributions by altering the amount of GeO${}_{2}$ and B${}_{2}$O${}_{3}$ doping on an FBG sensor. The strain and temperature sensitivities of the FBG can be varied by altering the amount of GeO${}_{2}$ and B${}_{2}$O${}_{3}$ doping [19]. The novelty of this method is due to its advantages in that it can be easily combined with OFDR–FBG systems, which can measure the Bragg wavelength distribution with high accuracy and a high spatial resolution of less than 1 mm [20], and the accuracy and stability are expected to be improved by increasing the number of fiber types with different doping levels. The validation and evaluation of the accuracy of the simultaneously measured distribution were conducted by using four-point bending tests in an environmental chamber. Furthermore, an error propagation analysis was applied to the experimental results, and measurement error factors are discussed.

## 2. Sensing System

### 2.1. Basic Idea of Simultaneous Measurement Using Doped FBGs

An FBG sensor is a diffraction grating system formed in an optical fiber. Refractive index modulation is observed by irradiating ultraviolet light onto GeO${}_{2}$ that is doped in an optical fiber core. An FBG sensor reflects light with the Bragg wavelength $\lambda_{B}$ corresponding to the grating period Λ and the effective refractive index *n*${}_{eff}$ of the core. This relationship is expressed as follows:

(1) $$\lambda_{B}=2n_{eff}\Lambda .$$

The grating period Λ and the effective refractive index *n*${}_{eff}$ vary with the strain and temperature. The Bragg wavelength shift $\Delta \lambda_{B}$ is calculated as follows:

(2) $${\Delta \lambda }_{B}=2n_{eff}\Lambda \left(1-\frac{{n_{eff}}^{2}}{2}\left[P_{12}-\nu \left(P_{11}+P_{12}\right)\right]\right)\Delta \epsilon_{fib}+2n_{eff}\Lambda \left[\alpha_{fib}+\frac{\left(\frac{dn}{dT}\right)}{n_{eff}}\right]\Delta T,$$

where *P*${}_{11}$ and *P*${}_{12}$ represent Pockel’s coefficients, ν denotes the Poisson ratio of the fiber, $\Delta \epsilon_{fib}$ denotes the strain of the fiber, $\alpha_{fib}$ denotes the coefficient of thermal expansion (CTE) of the fiber, and ΔT represents the temperature change. Here, the FBG sensor is bonded to the host material and measures the mechanical strain and thermal expansion of the host material. Therefore, $\Delta \epsilon_{fib}$ is expressed as follows:

(3) $$\Delta \epsilon_{fib}=\Delta \epsilon_{h\_mech}+\alpha_{h}\Delta T,$$

where $\Delta \epsilon_{h\_mech}$ and $\alpha_{h}$ denote the mechanical strain and CTE of the host material, respectively. From Equations (2) and (3), the Bragg wavelength shift relative to the change in the strain and temperature of the host material can be expressed as follows:

(4) $${\Delta \lambda }_{B}=K_{\epsilon }\Delta \epsilon_{h\_mech}+K_{T}\Delta T,$$

where

(5) $$K_{\epsilon }=2n_{eff}\Lambda \left(1-\frac{{n_{eff}}^{2}}{2}\left[P_{12}-\nu \left(P_{11}+P_{12}\right)\right]\right),$$

(6) $$K_{T}=2n_{eff}\Lambda \left(1-\frac{{n_{eff}}^{2}}{2}\left[P_{12}-\nu \left(P_{11}+P_{12}\right)\right]\right)\alpha_{h}+2n_{eff}\Lambda \left[\alpha_{fib}+\frac{\left(\frac{dn}{dT}\right)}{n_{eff}}\right].$$

**Figure 1:**
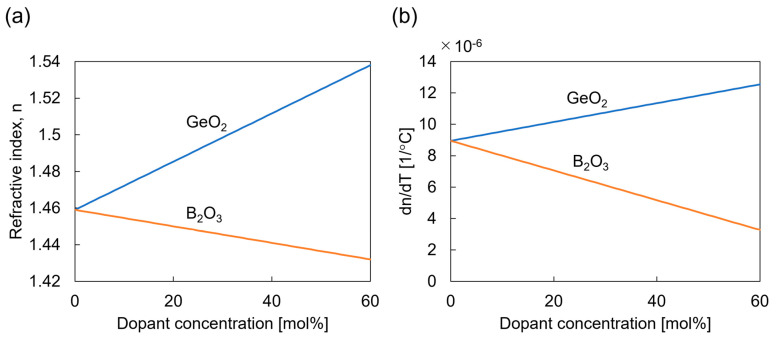
Variations in the respective parameters affecting the strain and temperature sensitivities as a function of the dopant concentration: (**a**) refractive index; (**b**) change rate of the refractive index with temperature.

As observed from Equations (5) and (6), the sensitivity to strain and temperature can be controlled by varying the effective refractive index $n_{eff}$ and the change rates of the refractive index with the temperature $\frac{dn}{dT}$. Doping GeO${}_{2}$ into the optical fiber core increases the effective refractive index and the change rates of the refractive index with the temperature, while B${}_{2}$O${}_{3}$ exhibits the opposite effect, as shown in Figure 1. By utilizing two or more FBG sensors with different GeO${}_{2}$ and B${}_{2}$O${}_{3}$ doping, the strain and temperature of the host material can be calculated as follows:

(7) $$\left(\begin{array}{c} {\Delta \lambda }_{B,1}\\ {\Delta \lambda }_{B,2}\\ \vdots \\ {\Delta \lambda }_{B,n} \end{array}\right)=\left(\begin{array}{cc} K_{\epsilon ,1} & K_{T,1}\\ K_{\epsilon ,2} & K_{T,2}\\ \vdots & \vdots \\ K_{\epsilon ,n} & K_{T,n} \end{array}\right)\left(\begin{array}{c} \Delta \epsilon_{h\_mech}\\ \Delta T \end{array}\right)=K\left(\begin{array}{c} \Delta \epsilon_{h\_mech}\\ \Delta T \end{array}\right).$$

(8) $$\left(\begin{array}{c} \Delta \epsilon_{h\_mech}\\ \Delta T \end{array}\right)=K^{-1}\left(\begin{array}{c} {\Delta \lambda }_{B,1}\\ {\Delta \lambda }_{B,2}\\ \vdots \\ {\Delta \lambda }_{B,n}. \end{array}\right).$$

In this study, four types of FBG sensors were used to validate the accuracy of simultaneous strain and temperature measurements.

### 2.2. Distributed Measurement Using an OFDR System

**Figure 2:**
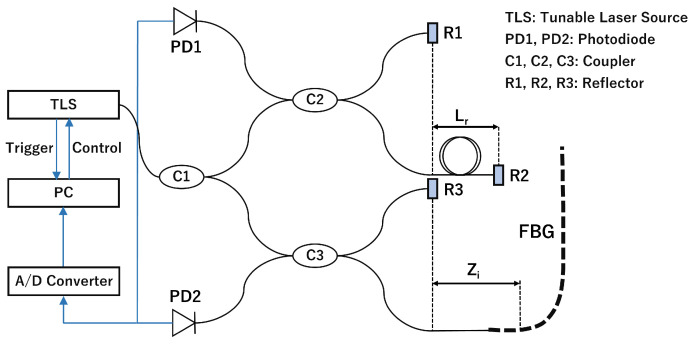
Schematic of the OFDR system used for distribution measurement.

A schematic of the OFDR system used for the distributed measurement is presented in Figure 2. The OFDR system comprised a tunable laser source (TLB-8800 Venturi^TM^, Newport Corp., California, USA) and two interferometers (LA-OFDR1500 C1, Lazoc Inc., Tokyo, Japan). Swept light entered each interferometer, with each returning a different interference signal. The reference interferometer returned an interference signal of the light reflected from Reflectors 1 and 2. The interference signal $I_{PD1}$ detected by Photodiode 1 is expressed as follows:

(9) $$I_{PD1}\propto cos\left(2n_{eff}L_{r}k\right),$$

where $L_{r}$ and *k* represent the distance between Reflectors 1 and 2 and the wavenumber, respectively. $L_{r}$ was set to 80 m in the system adopted in this study. This interference signal triggered the acquisition of the main interferometer signal. The main interferometer returned an interference signal of the reflected light from Reflector 3 and from each position of the FBG sensor. The interference signal $I_{PD2}$ detected by Photodiode 2 is expressed as follows:

(10) $$I_{PD2}\propto \sum R_{g}\left(z_{i},k\right)cos\left(2n_{eff}z_{i}k\right),$$

where $z_{i}$ and $R_{g}$ represent the position of the *i*-th grating and the reflection spectrum of the grating, respectively. By applying a short-time Fourier transform (STFT) to this signal, the intensity at each position and wavelength could be obtained. Therefore, the strain and temperature distributions could be calculated from the Bragg wavelength shift at each position in Equation (8).

## 3. Materials and Methods

**Figure 3:**
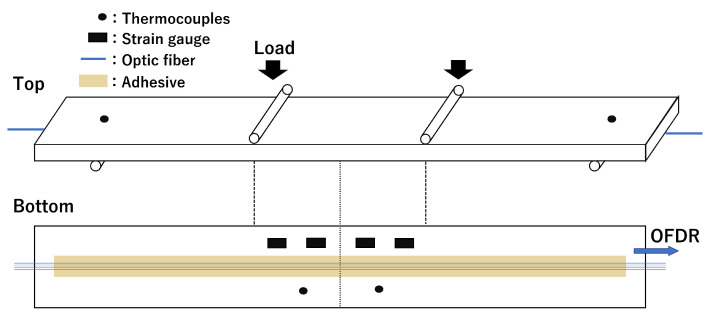
Schematic of the four-point bending test.

**Figure 4:**
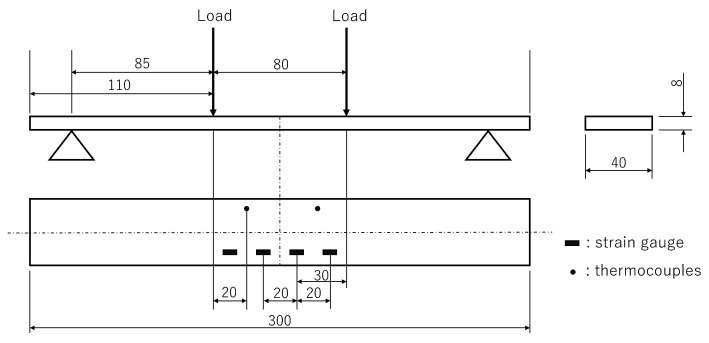
Geometry of the specimen used for the four-point bending test.

A schematic of the four-point bending test is presented in Figure 3. An SUS 304 coupon was prepared with a length, width, and thickness of 300, 40, and 8 mm, respectively. Four types of optical fibers with different GeO${}_{2}$ and B${}_{2}$O${}_{3}$ doping (F1: high-NA fiber, F2: ultraviolet transparent fiber, F3: normal single-mode fiber, F4: photosensitive fiber, Fujikura Ltd., Tokyo, Japan) were used. All fibers were 150 μm in diameter and had an FBG length of 100 mm. These fibers were bonded to the bottom side of the specimen by using an epoxy adhesive (NP-50, Tokyo Sokki Kenkyujo Co., Ltd., Tokyo, Japan). Strain gauges and thermocouples were also placed on the specimen for comparisons with the fiber-optic measurements. Four strain gauges were bonded to the center of the bottom side of the specimen at 20 mm intervals, as illustrated in Figure 4. Thermocouples were taped on the specimen, with two on the top side and two on the bottom side. The fiber-optic measurement conditions were as follows: wavelength sweep range: 1548–1568 nm, sampling rate: approximately 10 Hz, STFT window length: 400 pm, wavelength resolution: 5 pm, and position resolution: 1 mm.

**Figure 5:**
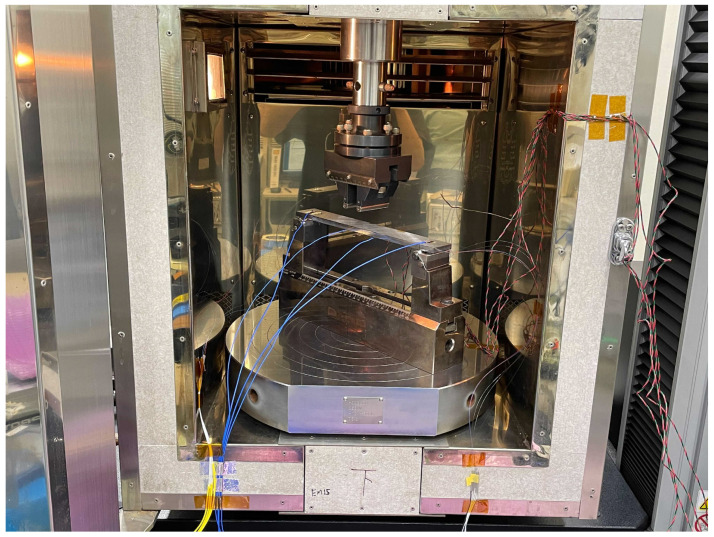
Specimen placed in an environmental chamber.

The four-point bending loads were applied to the top side of the specimen with an 80 mm interval. Two supporting bars were placed on the bottom side of the specimen with a 250 mm interval. As illustrated in Figure 5, the specimen was placed in an environmental chamber to allow for temperature changes. First, to obtain the strain sensitivity, loads of approximately 0, 250, 500, 750, 1000, 1250, and 1500 N were applied at 25 °C. The Bragg wavelength distribution was measured at each stage of the strain. Similarly, to obtain the temperature sensitivity, the temperature was set to 25, 50, 75, and 100 °C in the unloaded condition. The Bragg wavelength distribution was measured after confirming that the temperature was uniform and stable. After obtaining the strain and temperature sensitivity, simultaneous measurements of strain and temperature were conducted. The temperature was set to 25, 50, 75, and 100 °C, and loads of approximately 0, 250, 500, 750, 1000, 1250, and 1500 N were applied for each temperature case. Fiber-optic measurements were performed five times for each of the test cases.

## 4. Results

### 4.1. Strain and Temperature Sensitivity

**Figure 6:**
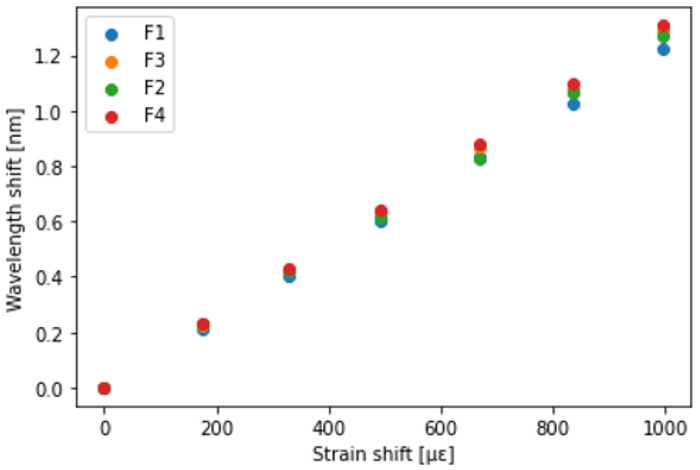
Bragg wavelength shift as a function of strain.

**Figure 7:**
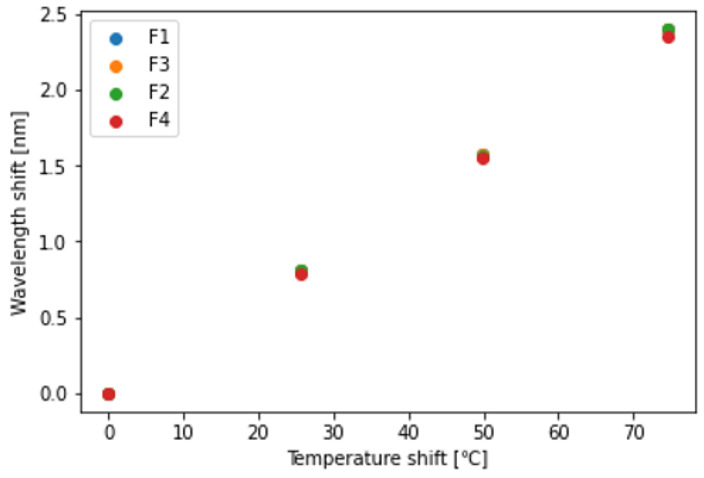
Bragg wavelength shift as a function of temperature.

Figure 6 and Figure 7 present the Bragg wavelength shift as a function of strain and temperature. The Bragg wavelength shift was spatially averaged over a 60 mm section in the middle of the specimen where the strain and temperature were uniformly distributed. It was verified that the Bragg wavelength shifted linearly with the change in strain and temperature. Each fiber exhibited a different sensitivity to strain and temperature. Regarding the strain sensitivity, F4 exhibited the highest sensitivity and F1 exhibited the lowest sensitivity. Conversely, F1 exhibited the highest temperature sensitivity and F4 exhibited the lowest temperature sensitivity. The fact that the strain sensitivity trend differed from the temperature sensitivity trend was a desirable characteristic for their simultaneous measurement. The sensitivities $K_{\epsilon }$ and $K_{T}$ of each fiber are summarized in Table 1.

### 4.2. Simultaneous Measurement

The root-mean-squared error (RMSE) was adopted to evaluate the error of the simultaneous strain and temperature measurements. The RMSE is expressed as follows:

(11) $$error=\sqrt{\frac{1}{n}\sum_{k=0}^{n}{\left(y_{estimation,k}-{\hat{y}}_{reference,k}\right)}^{2}},$$

where $y_{estimation}$ is the estimated value obtained by adopting Equation (8), ${\hat{y}}_{reference}$ is the reference value obtained from the strain gauges or thermocouples, and *k* denotes a test case.

Table 2 summarizes the errors of the simultaneous strain and temperature measurements when the two types of fibers were utilized. Combinations of fibers with close sensitivities, such as F2 and F3, resulted in a significant strain error of 287.2 μϵ and a temperature error of 11.70 °C. The best combination was the one with the largest sensitivity difference, i.e., the combination of F1 and F4, with the strain and temperature errors of 34.1 μϵ and 1.74 °C, respectively. Table 3 presents the errors of simultaneous measurements when three or four types of fibers were utilized. The combination of three types of fibers stabilized the accuracy of simultaneous measurements, and even the worst combination of F2, F3, and F4 resulted in strain and temperature errors of 103.2 μϵ and 4.31 °C, respectively. The combination including both F1 and F4 exhibited the lowest error, and the best combination of F1, F2, and F4 exhibited strain and temperature errors of 28.4 μϵ and 1.52 °C, respectively. The combination of the four types of fibers did not exhibit any improvement in accuracy compared to the combinations of three types of fibers.

**Figure 8:**
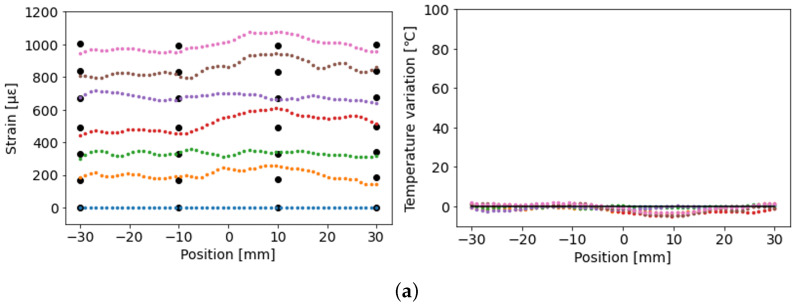

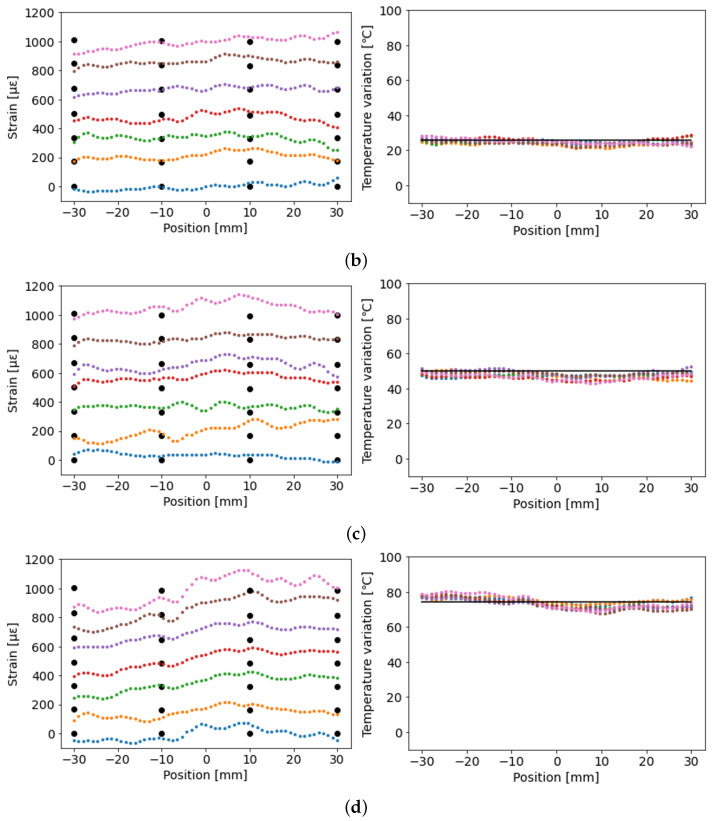
Strain and temperature distributions measured by combining F1, F2, and F4: (**a**) 25 °C case (ΔT=0); (**b**) 50 °C case (ΔT=25); (**c**) 75 °C case (ΔT=50); (**d**) 100 °C case (ΔT=75).

Figure 8 presents the strain and temperature distributions measured by combining F1, F2, and F4. Position zero corresponds to the center of the specimen. The black dots in the strain distribution graph represent the values measured by using the strain gauges. The black line in the graph of the temperature distribution represents the average value measured by the four thermocouples. It was inferred that uniform strain and temperature distributions were measured over a 60 mm section, which agreed well with the values of the strain gauges and thermocouples.

## 5. Discussion

Simulations with an error propagation model were conducted to evaluate the errors in the simultaneous strain and temperature measurements. The separation of strain and temperature by using two types of fibers can be achieved by solving the following simultaneous equations.

(12) $$\left\{\begin{array}{c} \Delta \lambda_{B,1}=K_{\epsilon ,1}\Delta \epsilon +K_{T,1}\Delta T.\\ \Delta \lambda_{B,2}=K_{\epsilon ,2}\Delta \epsilon +K_{T,2}\Delta T. \end{array}\right.$$

Δλ, $K_{\epsilon }$, and $K_{T}$ are measured values. Therefore, these values include errors due to the instrument, misalignment of measurement positions, etc. The errors in strain and temperature are considered to be triggered by the propagation of errors in the three aforementioned parameters. Accordingly, the following assumptions are made.

1. Errors are due to measurement errors in Δλ, $K_{\epsilon }$, and $K_{T}$.
2. The error amount for each error factor is the same value for all fibers.
3. Each error occurs independently and follows a normal distribution.

**Figure 9:**
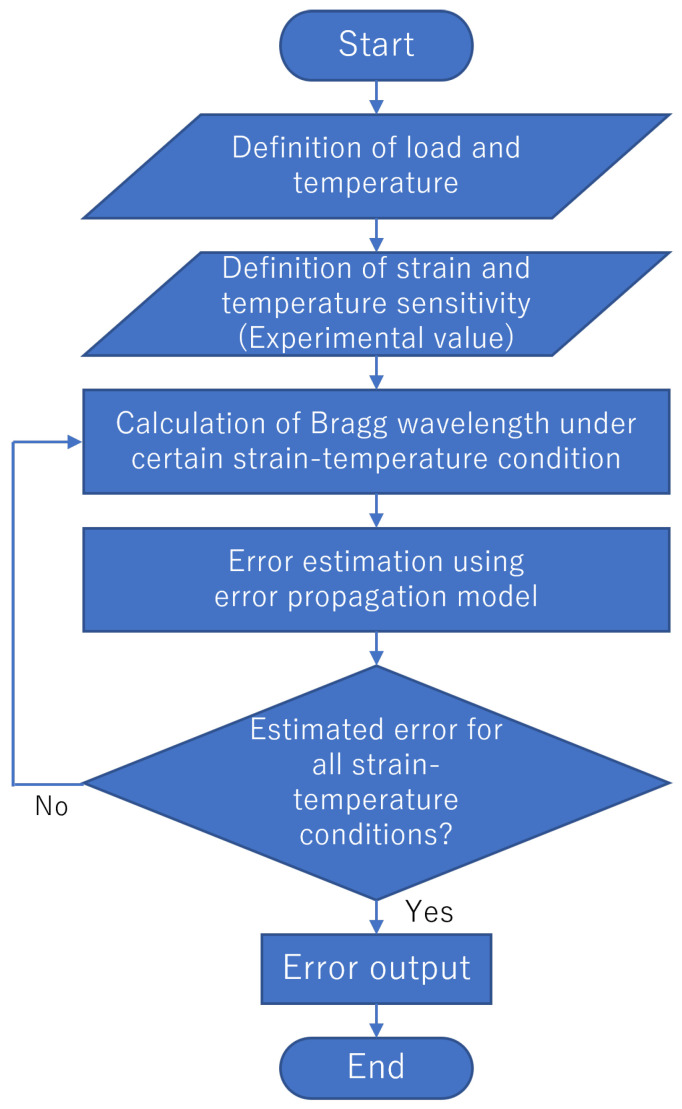
Flow of the error output when using an error propagation model.

**Figure 10:**
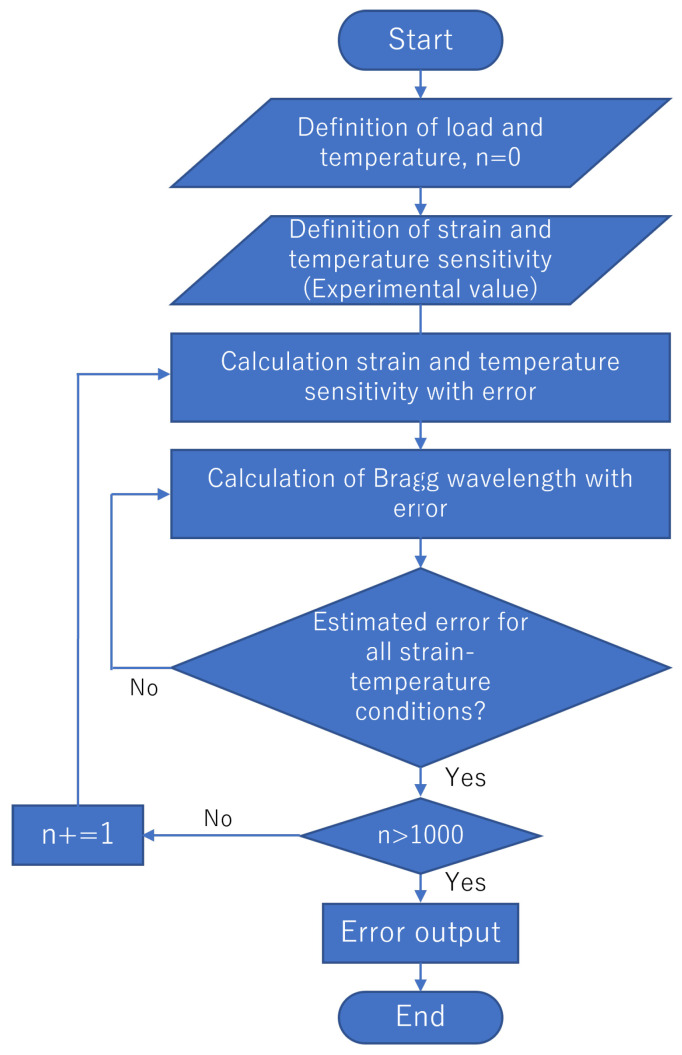
Flow of the error output when using the Monte Carlo simulation.

Although the sensitivity characteristics of the four types of fibers differed, their geometry and handling conditions were all the same. Therefore, Assumption 2 appears logical. Assumption 3 also appears logical because the strain and temperature sensitivities were determined from independent tests. With these three assumptions, the strain $e_{\Delta \epsilon }$ and temperature $e_{\Delta T}$ errors can be defined by the following equation of the error propagation law:

(13) $$e_{\Delta \epsilon }=\sqrt{{\left(\frac{\partial \Delta \epsilon }{\partial K_{\epsilon }}\right)}^{2}e_{K_{\epsilon }}^{2}+{\left(\frac{\partial \Delta \epsilon }{\partial K_{T}}\right)}^{2}e_{K_{T}}^{2}+{\left(\frac{\partial \Delta \epsilon }{\partial \Delta \lambda_{B}}\right)}^{2}e_{\Delta \lambda_{B}}^{2}},$$

(14) $$e_{\Delta T}=\sqrt{{\left(\frac{\partial \Delta T}{\partial K_{\epsilon }}\right)}^{2}e_{K_{\epsilon }}^{2}+{\left(\frac{\partial \Delta T}{\partial K_{T}}\right)}^{2}e_{K_{T}}^{2}+{\left(\frac{\partial \Delta T}{\partial \Delta \lambda_{B}}\right)}^{2}e_{\Delta \lambda_{B}}^{2}},$$

where $e_{K_{\epsilon }}$, $e_{K_{T}}$, and $e_{\Delta \lambda_{B}}$ represent the values of the estimated error for $K_{\epsilon }$, $K_{T}$, and $\Delta \lambda_{B}$, respectively. In addition, $e_{K_{\epsilon }}$ and $e_{K_{T}}$ denote the standard deviations of the strain and temperature sensitivities for the three tests in Section 4.1. $\Delta \lambda_{B}$ represents the standard deviation of five measurements of the Bragg wavelength averaged over a 60 mm section with no load applied. The values for each estimated error are summarized in Table 4. The flow of the simulation using the error propagation model is illustrated in Figure 9, and the calculated errors are presented in Table 5. Monte Carlo simulations were also performed to evaluate the error when more than three fibers were utilized. The parameters in Table 4 were given as a normal distribution in the simulation. The flow of the Monte Carlo simulation is illustrated in Figure 10, and the calculated errors are presented in Table 6. The simulations with F1, F3, and F4 (F1&F3, F1&F4, F3&F4, and F1&F3&F4) generally agreed well with the experiments, thereby indicating the validity of the simulations with the error propagation model. The differences observed in the experiments might have been due to the influence of the measurement accuracy, which depended on the adhesion conditions and the misalignment of the measurement position with the sensors that were used for the correct values. For combinations involving the F2 fiber, the error was more significant in the experiments than in the simulations. It is suggested that the strain transfer of the host material may have differed due to the effect of adhesion.

## 6. Conclusions

We proposed a method for the simultaneous measurement of strain and temperature by varying the GeO${}_{2}$ and B${}_{2}$O${}_{3}$ doping in optical fibers. To verify the accuracy of the measurements, we conducted four-point bending tests in an environmental chamber with four types of fibers. The optimal combination of sensors resulted in strain and temperature errors of 28.4 μϵ and of 1.52 °C, respectively, thereby demonstrating the effectiveness of the proposed method. Furthermore, we discussed the sources of error by using an error propagation analysis. The results of the error propagation analysis agreed well with the experimental results, thus indicating the effectiveness of the analysis for verifying accuracy and determining the factors affecting accuracy. To the best of our knowledge, this is the first time in the literature that error propagation analysis has been applied to the simultaneous measurement of strain and temperature by using optical fibers, and we expect accuracy verification to be easier in the future.

## Figures and Tables

**Table 1:** Sensitivity of each fiber.

	F1	F2	F3	F4
K_ϵ [pm/μϵ]	1.2350	1.2691	1.2968	1.3154
K_T [pm/°C]	32.106	32.010	32.072	31.482

**Table 2:** Evaluated error of simultaneous measurements when combining two types of fibers.

	F1&F2	F1&F3	F1&F4	F2&F3	F2&F4	F3&F4
Error of strain [μϵ]	252.5	75.6	34.1	287.2	117.2	47.6
Error of temp. [°C]	9.82	3.31	1.74	11.70	4.73	1.58

**Table 3:** Evaluated error of simultaneous measurement when combining three or four types of fibers.

	F1&F2&F3	F1&F2&F4	F1&F3&F4	F2&F3&F4	F1&F2&F3&F4
Error of strain [μϵ]	73.7	28.4	36.2	103.2	31.1
Error of temp. [°C]	3.24	1.52	1.82	4.31	1.64

**Table 4:** Estimated error for each error factor.

$e_{K_{\epsilon }}$ [pm/μϵ]	$e_{K_{T}}$ [pm/°C]	$e_{\lambda_{B}}$ [pm]
$1.59\times 10^{-3}$	$5.59\times 10^{-2}$	$1.00\times 10^{-2}$

**Table 5:** Calculated errors when using the error propagation model.

	F1&F2	F1&F3	F1&F4	F2&F3	F2&F4	F3&F4
Error of strain [μϵ]	105.4	62.2	37.2	155.5	57.9	92.6
Error of temp. [°C]	4.10	2.47	1.51	6.25	2.37	3.80

**Table 6:** Calculated errors when using the Monte Carlo simulation.

	**F1&F2**	**F1&F3**	**F1&F4**	**F2&F3**	**F2&F4**	**F3&F4**
Error of strain [μϵ]	33.7	30.6	21.2	312.4	33.5	33.3
Error of temp. [°C]	1.41	1.41	0.89	12.60	1.42	1.34
	**F1&F2&F3**	**F1&F2&F4**	**F1&F3&F4**	**F2&F3&F4**	**F1&F2&F3&F4**	
Error of strain [μϵ]	59.6	21.9	21.9	46.0	53.3	
Error of temp. [°C]	2.34	0.86	1.12	1.88	2.09	

## Data Availability

Not applicable.

## References

[1] López-Higuera J.M., Cobo L.R., Incera A.Q., Cobo A. (2011). Fiber Optic Sensors in Structural Health Monitoring. J. Light. Technol..

[2] Wang H., Jiang L., Xiang P. (2018). Improving the durability of the optical fiber sensor based on strain transfer analysis. Opt. Fiber Technol..

[3] Xu M.G., Archambault J.L., Reekie L., Dakin J.P. (1994). Discrimination between strain and temperature effects using dual-wavelength fibre grating sensors. Electron. Lett..

[4] Patrick H.J., Williams G.M., Kersey A.D., Pedrazzani J.R., Vengsarkar A.M. (1996). Hybrid fiber Bragg grating/long period fiber grating sensor for strain/temperature discrimination. IEEE Photonic Technol. Lett..

[5] Guan B.O., Tam H.Y., Tao X.M., Dong X.Y. (2000). Simultaneous strain and temperature measurement using a superstructure fiber Bragg grating. IEEE Photonic Technol. Lett..

[6] Mondal S.K., Tiwari U., Poddar G.C., Mishra V., Singh N., Jain S.C., Sarkar S.N., Chattoypadhya K.D., Kapur P. (2009). Single fiber Bragg grating sensor with two sections of different diameters for longitudinal strain and temperature discrimination with enhanced strain sensitivity. Rev. Sci. Instrum..

[7] Zhang N., Xu W., You S., Yu C., Yu C., Dong B. (2018). Simultaneous measurement of refractive index, strain and temperature using a tapered structure based on SMF. Opt. Commun..

[8] Sarkar S., Tarhani M., Eghbal M.K., Shadaram M. (2020). Discrimination between strain and temperature effects of a single fiber Bragg grating sensor using sidelobe power. J. Appl. Phys..

[9] Takeda S., Sato M., Ogasawara T. (2022). Simultaneous measurement of strain and temperature using a tilted fiber Bragg grating. Sens. Actuators A-Phys..

[10] Kikuchi M., Ogasawara T., Fujii S., Takeda S. (2022). Application of machine learning for improved accuracy of simultaneous temperature and strain measurements of carbon fiber-reinforced plastic laminates using an embedded tilted fiber Bragg grating sensor. Compos. Part A-Appl. Sci..

[11] Zhou D.P., Li W., Chen L., Bao X. (2013). Distributed temperature and strain discrimination with stimulated Brillouin scattering and Rayleigh backscatter in an optical fiber. Sensors.

[12] Wada D., Igawa H., Murayama H. (2016). Simultaneous distributed measurement of the strain and temperature for a four-point bending test using polarization-maintaining fiber Bragg grating interrogated by optical frequency domain reflectometry. Measurement.

[13] Zhu M., Murayama H., Wada D. (2017). Self-Evaluation of PANDA-FBG Based Sensing System for Dynamic Distributed Strain and Temperature Measurement. Sensors.

[14] Soto M.A., Thévenaz L. (2013). Modeling and evaluating the performance of Brillouin distributed optical fiber sensors. Opt. Express.

[15] Murayama H., Kageyama K., Uzawa K., Ohara K., Igawa H. (2012). Strain monitoring of a single-lap joint with embedded fiber-optic distributed sensors. Struct. Health Monit..

[16] Mekid S., Vacharanukul K. (2006). Differential Laser Doppler based Non-Contact Sensor for Dimensional Inspection with Error Propagation Evaluation. Sensors.

[17] Sanders-Reed J.N. (2001). Error propagation in two-sensor three-dimensional position estimation. Opt. Eng..

[18] Hartzell P.J., Gadomski P.J., Glennie C.L., Finnegan D.C., Deems J.S. (2015). Rigorous error propagation for terrestrial laser scanning with application to snow volume uncertainty. Struct. Health Monit..

[19] Han Y.G., Lee S.B., Kim C.S., Kang J.U., Paek U.C., Chung Y. (2003). Simultaneous measurement of temperature and strain using dual long-period fiber gratings with controlled temperature and strain sensitivities. Opt. Express.

[20] Igawa H., Ohta K., Kasai T., Yamaguchi I., Murayama H., Kageyama K. (2008). Distributed measurements with a long gauge FBG sensor using optical frequency domain reflectometry (1st report, system investigation using optical simulation model). J. Solid Mech. Mater. Eng..

